# Swedish Cure for Drunkenness

**Published:** 1892-05

**Authors:** 


### THE SWEDISH CURE FOR DRUNKENNESS.

The habitual drunkard in Norway or Sweden renders himself liable to imprisonment for his love of strong drink, and during his incarceration he is required to submit to a plan of treatment for the cure of his failing which is said to produce marvelous results. The plan, says the "Family Doctor," consists in making the delinquent subsist entirely on bread and wine. The bread is steeped in a bowl of wine for an hour or more before the meal is served. The first day the habitual toper takes his food in this shape without any repugnance ; the second day he fiinds it less agreeable to his palate ; finally, he positively loathes the sight of it. Experience shows that a period of from eight to ten days of this regime is generally more than sufficient to make a man evince the greatest aversion to any thing in the shape of wine. Many men, after their incarceration, become total abstainers.

